# Inhibiting NFAT1 for breast cancer therapy: New insights into the mechanism of action of MDM2 inhibitor JapA

**DOI:** 10.18632/oncotarget.5851

**Published:** 2015-10-08

**Authors:** Jiang-Jiang Qin, Wei Wang, Sukesh Voruganti, Hui Wang, Wei-Dong Zhang, Ruiwen Zhang

**Affiliations:** ^1^ Department of Pharmaceutical Sciences, School of Pharmacy, Texas Tech University Health Sciences Center, Amarillo, TX, USA; ^2^ Cancer Biology Center, School of Pharmacy, Texas Tech University Health Sciences Center, Amarillo, TX, USA; ^3^ Institute for Nutritional Sciences, Shanghai Institutes for Biological Sciences, Chinese Academy of Sciences, Shanghai, PR China; ^4^ School of Pharmacy, Shanghai Jiao Tong University, Shanghai, PR China

**Keywords:** JapA, NFAT1, MDM2, p53, breast cancer

## Abstract

Transcription factor NFAT1 has been recently identified as a new regulator of the *MDM2* oncogene. Targeting the NFAT1-MDM2 pathway represents a novel approach to cancer therapy. We have recently identified a natural product MDM2 inhibitor, termed JapA. As a specific and potent MDM2 inhibitor, JapA inhibits MDM2 at transcriptional and post-translational levels. However, the molecular mechanism remains to be fully elucidated for its inhibitory effects on *MDM2* transcription. Herein, we reported that JapA inhibited NFAT1 and NFAT1-mediated *MDM2* transcription, which contributed to the anticancer activity of JapA. Its effects on the expression and activity of NFAT1 were examined in various breast cancer cell lines *in vitro* and in MCF-7 and MDA-MB-231 xenograft tumors *in vivo*. The specificity of JapA in targeting NFAT1 and NFAT1-MDM2 pathway and the importance of NFAT1 inhibition in JapA's anticancer activity were demonstrated using NFAT1 overexpression and knockdown cell lines and the pharmacological activators and inhibitors of NFAT1 signaling. Our results indicated that JapA inhibited NFAT1 signaling in breast cancer cells *in vitro* and *in vivo*, which plays a pivotal role in its anticancer activity. JapA inhibited the nuclear localization of NFAT1, disrupted the NFAT1-*MDM2* P2 promoter complex, and induced NFAT1 proteasomal degradation, resulting in the repression of *MDM2* transcription. In conclusion, JapA is a novel NFAT1 inhibitor and the NFAT1 inhibition is responsible for the JapA-induced repression of *MDM2* transcription, contributing to its anticancer activity. The results may pave an avenue for validating the NFAT1-MDM2 pathway as a novel molecular target for cancer therapy.

## INTRODUCTION

**Figure 1:**
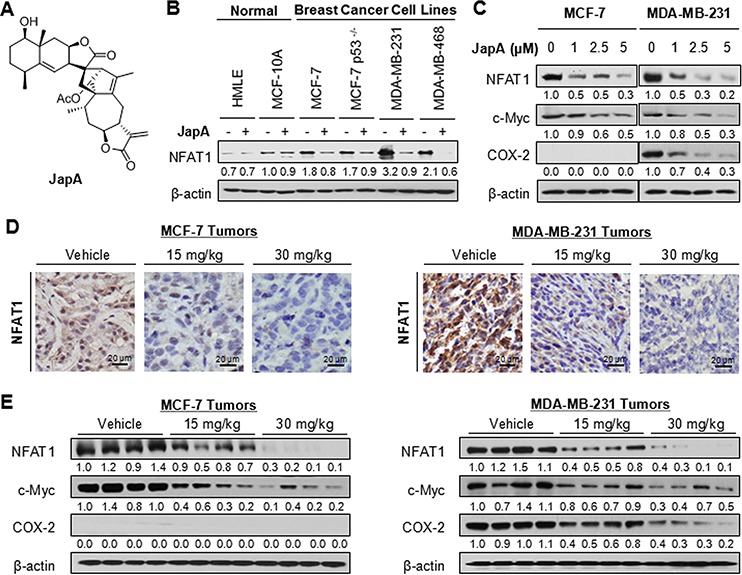
Effects of JapA on NFAT1 signaling pathway in breast cancer cells *in vitro* and *in vivo* **A.** The chemical structure of JapA. **B.** Human breast epithelial cells and breast cancer cells were exposed to 2 μM JapA for 24 h. The protein level of NFAT1 was detected by western blotting. **C.** MCF-7 and MDA-MB-231 cells were exposed to various concentrations of JapA for 24 h for the protein expression of NFAT1, c-Myc and COX-2. The experiments were repeated three times. **D, E.** JapA was administered by i.p. injection (15 or 30 mg/kg/d, 5 d/wk) to nude mice bearing MCF-7 or MDA-MB-231 xenograft tumors. After 5 weeks (MCF-7) or 3 weeks (MDA-MB-231) JapA treatment, the tumor tissues were analyzed for (D) the protein expression of NFAT1 by immunohistochemistry (scale bar, 20 μm) and (E) the protein expression of NFAT1, c-Myc and COX-2 by Western blotting (each lane represents a different tumor sample). The intensity ratio under each band was obtained by IMAGEJ software analysis normalized on untreated control.

Oncogene addiction demonstrates the dependence of cancer cells on a single or few activated oncogenes for their survival, which has been supported by the accumulating evidence from preclinical and clinical studies [1–2]. Therefore, treating the specific type of cancer via the inhibition of corresponding oncogenes can lead to dramatic response and improved survival of patients with cancer [2]. The mouse double minute 2 (*MDM2*) oncogene is amplified and overexpressed in various human cancers and plays an important role in cancer development and progression via p53-dependent and p53-independent mechanisms of action [3–7]. MDM2 has been demonstrated as a promising molecular target for cancer therapy and many MDM2 inhibitors have been developed as new anticancer drugs [8–15]. In an effort to screen small molecule inhibitors of MDM2 for the development of novel and effective cancer therapeutics, we identified a potent and selective MDM2 inhibitor, termed JapA (Figure 1A) [13].

JapA is a structurally unique dimeric sesquiterpenoid that was isolated from the aerial part of *Inula japonica* Thunb, a plant that has been used in traditional Chinese medicine for the treatment of inflammation, diabetes, digestive disorders, and bronchitis [16–18]. JapA has the similar structural features of artemisinin and parthenolide, which are under preclinical and clinical studies for cancer therapy [19]. However, due to its dimerization status and unique mechanisms of action, JapA is considered to be more effective than these analogs as an anticancer drug. We have demonstrated that JapA inhibits the tumor growth and prevents metastasis in breast cancer xenograft models, without inducing any host toxicity [13]. It has also been observed that JapA is safe and effective in treating other human cancers containing high expression levels of MDM2, *e.g.*, Burkitt lymphoma and lung cancer [20–21]. Mechanistically, JapA decreases MDM2 protein stability and inhibits *MDM2* transcription, regardless of p53 status of the cells or tumors [13]. At the post-translational level, JapA directly binds to MDM2 protein and induces MDM2 auto-ubiquitination and proteasomal degradation. JapA has also been found to inhibit *MDM2* transcription in a nuclear factor of activated T cells (NFAT)-dependent manner, but the molecular mechanism is still not clear yet.

NFAT is a group of inducible transcription factors with five distinct family members NFAT1 to NFAT5, and it has been demonstrated to play crucial roles in the regulation of various aspects of the immune system and numerous developmental programs in vertebrates (reviewed in references [22–23]). The NFAT proteins regulate diverse cellular functions, such as cell survival, cell cycle progression, migration, invasion, and angiogenesis [24–25]. Increasing evidence suggests the dual roles for NFAT isoforms as oncogene and tumor suppressor in different types of human cancer [24, 26]. NFAT1, the first identified member of NFAT family is overexpressed and constitutively activated in several human cancers, including breast cancer [27–30]. NFAT1 is involved in the tumor growth and metastasis through regulating the expression of its target genes, *e.g.*, c-Myc and Cyclooxygenase 2 (COX-2) [24–26]. We have recently discovered that NFAT1 activates the *MDM2* oncogene and this pathway contributes to the overexpression of MDM2 in cancer cells with non-functional p53 [31]. Therefore, targeting NFAT1 and NFAT1-MDM2 pathway could be a promising strategy for the discovery of novel cancer therapeutic agents.

The present study was designed to investigate the molecular mechanisms for NFAT1-mediated inhibitory effects of JapA on *MDM2* transcription and to demonstrate the role of NFAT1 in JapA's anticancer activity *in vitro* and *in vivo*. Since the initial evidence for JapA-induced MDM2 inhibition was obtained with *in vitro* and *in vivo* breast cancer models [13], we utilized the same models in the present study. Our results not only helped elucidate the molecular mechanism of JapA as a new class of NFAT1 inhibitor, but also would facilitate the validation of the therapeutic potential of targeting NFAT1 and NFAT1-MDM2 pathway, providing a basis for further preclinical and clinical development of NFAT1-MDM2 inhibitors for human cancer therapy.

## RESULTS

### JapA inhibits NFAT1 signaling in breast cancer cells *in vitro* and *in vivo*

JapA has been found to inhibit the *MDM2* transcription in an NFAT-dependent manner, while NFAT1 has been recently identified as a novel activator of the *MDM2* oncogene [13, 31]. Therefore, we examined whether JapA (Figure 1A) affects NFAT1 expression in human normal breast cells and breast cancer cells. As shown in Figure 1B, a significant inhibition of NFAT1 expression by JapA was observed in MCF-7 (p53 wild-type), MCF-7/p53^−/−^ (p53 knockdown), MDA-MB-231 (p53 mutant), and MDA-MB-468 (p53 mutant) human breast cancer cell lines. There was no apparent reduction of NFAT1 expression levels in human breast epithelial MCF-10A and human mammary luminal epithelial (HMLE) cell lines. We further demonstrated that JapA inhibited the protein expression of NFAT1 and its transcriptional responsive genes c-Myc and COX-2 in a concentration-dependent manner in both the MCF-7 and MDA-MB-231 cell lines (Figure 1C).

The effects of JapA on the NFAT1 signaling were examined in the same breast cancer xenograft tumors we used in the previous study [13]. The significant downregulation of MDM2 expression levels by JapA has been observed in these tumor samples. Compared with vehicle-treated tumors, JapA reduced the expression levels of NFAT1 in both the MCF-7 and MDA-MB-231 breast cancer xenograft models, as detected by immunohistochemical staining (Figure 1D) and western blotting (Figure 1E). Consistent with the *in vitro* results, JapA treatment also reduced the protein levels of c-Myc and COX-2 in the tumors (Figure 1E). Together, these results suggested that JapA inhibits NFAT1 signaling in breast cancer cells *in vitro* and *in vivo*.

### JapA inhibits NFAT1-mediated MDM2 protein expression

**Figure 2:**
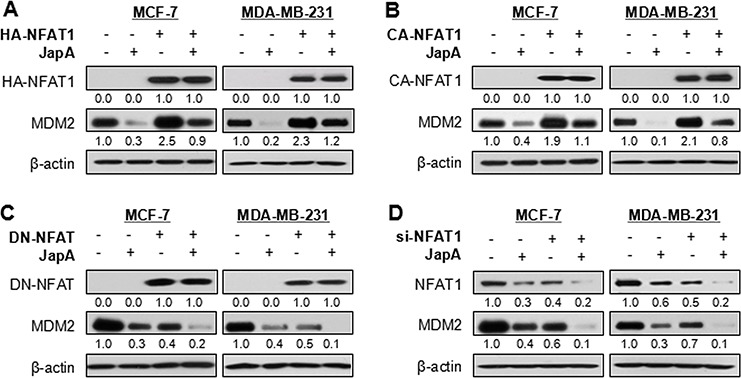
Effects of JapA on NFAT1-mediated MDM2 protein expression **A, B.** MCF-7 and MDA-MB-231 cells were transfected with (A) a HA-tagged NFAT1 plasmid or (B) a HA-tagged constitutively activated NFAT1 (CA-NFAT1) plasmid, followed by treatment with JapA (1 and 2 μM, respectively, for MCF-7 and MDA-MB-231 cells) for 24 h. The NFAT1 protein levels were detected by Western blotting using antibody against HA. **C, D.** The cells were transfected with a FLAG-tagged dominant-negative NFAT (DN-NFAT) plasmid, or with NFAT1 siRNA (si-NFAT1), followed by treatment with JapA for 24 h. The NFAT1 protein levels were detected by Western blotting using antibodies against FLAG (C) or NFAT1 (D), respectively. The intensity ratio under each band was obtained by IMAGEJ software analysis normalized on untreated control. All of the experiments were repeated three times.

Considering that NFAT1 upregulates MDM2 expression in a p53-independent manner [31], we examined the effects of JapA on NFAT1-mediated MDM2 expression. As shown in Figure 2A, JapA decreased the MDM2 protein expression in both MCF-7 and MDA-MB-231 cell lines, regardless of p53 status of the cells. However, the enforced expression of NFAT1 induced MDM2 protein expression and reduced the inhibition of MDM2 by JapA. Similar results were observed in breast cancer cells transfected with or without HA-tagged constitutively-activated NFAT1 (CA-NFAT1) followed by JapA treatment (Figure 2B). In contrast, the transfection of a dominant-negative NFAT (DN-NFAT) (Figure 2C) or NFAT1 siRNA (Figure 2D) led to the downregulation of MDM2 expression, which was further enhanced by JapA in both breast cancer cell lines. These results indicated an inhibitory effect of JapA on NFAT1-activated MDM2 protein expression.

### JapA represses NFAT1-mediated *MDM2* transcription

**Figure 3:**
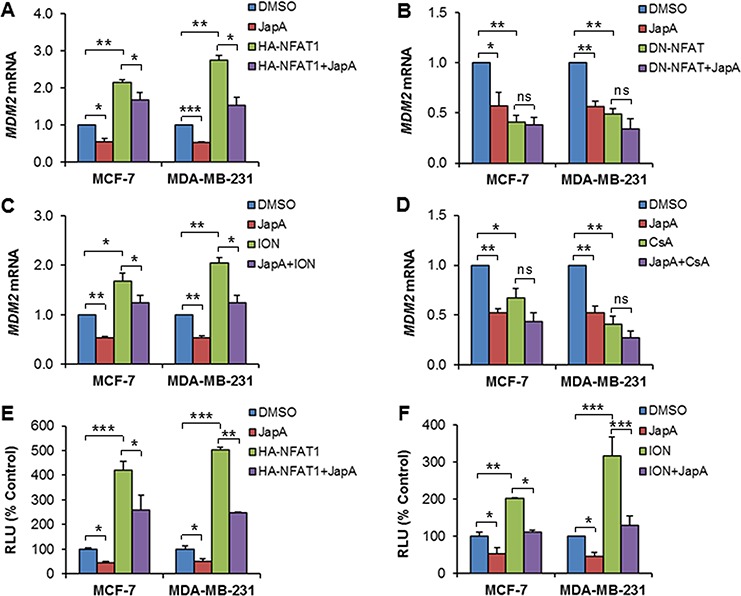
Effects of JapA on NFAT1-mediated *MDM2* transcription **A, B.** MCF-7 and MDA-MB-231 cells were transfected with (A) an HA-tagged NFAT1 plasmid or (B) a FLAG-tagged dominant-negative NFAT (DN-NFAT) plasmid, followed by treatment with JapA (1 and 2 μM, respectively, for MCF-7 and MDA-MB-231 cells) for 24 h. The relative mRNA levels of *MDM2* were determined by real-time quantitative PCR. **C, D.** The cells were treated with JapA in the presence or absence of (C) ionomycin (ION; 4 μM) or (D) cyclosporine A (CsA; 2 μM) for 24 h. The relative mRNA levels of *MDM2* were determined by real-time quantitative PCR. **E, F.** The cells were transfected with a HA-NFAT1 plasmid combined with the full-length (Luc 01) *MDM2* P2 promoter (E), or with the full-length (Luc 01) *MDM2* P2 promoter alone (F), followed by treatment with JapA (E), or JapA with or without ION (4 μM) (F) for 24 h. The MDM2 luciferase activity was determined using the Dual-Luciferase Reporter Assay System. All of the experiments were repeated three times. (*P < 0.05, **P < 0.01, and ***P < 0.001, “ns” denotes “not significant”).

We further examined the effects of JapA on NFAT1-mediated *MDM2* transcription. As shown in Figure 3A, HA-NFAT1 overexpression increased the *MDM2* mRNA levels (*P* < 0.01), and JapA reduced both the basic and HA-NFAT1-induced *MDM2* mRNA expression (*P* < 0.05) in both MCF-7 and MDA-MB-231 cell lines. However, JapA treatment had no significant effects on the DN-NFAT-attenuated *MDM2* mRNA expression in these cells (Figure 3B). Similarly, JapA reduced the *MDM2* mRNA expression that was induced by ionomycin (ION, an activator of calcineurin) (Figure 3C), but it did not show significant enhancement of the downregulation of the *MDM2* mRNA levels by cyclosporine A (CsA, a calcineurin inhibitor) (Figure 3D). Furthermore, enforced NFAT1 expression increased the MDM2 luciferase activity (*P* < 0.001), which was reduced by JapA treatment (*P* < 0.05) (Figure 3E). Similar results were observed when NFAT1 was activated using ION (Figure 3F).

### JapA inhibits the nuclear localization of NFAT1

**Figure 4:**
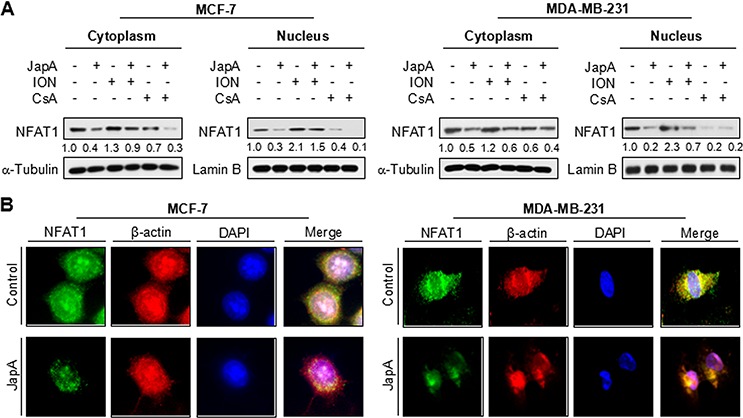
Effects of JapA on the nuclear localization of NFAT1 **A.** MCF-7 and MDA-MB-231 cells were treated with JapA (1 and 2 μM, respectively) in the presence or absence of ionomycin (ION; 4 μM) or cyclosporine A (CsA; 2 μM) for 24 h. The nuclear and cytosolic proteins were extracted and examined by Western blotting. Lamin B and α-tubulin were used as the internal references, respectively. The intensity ratio under each band was obtained by IMAGEJ software analysis normalized on untreated control. **B.** The cells were treated with JapA or vehicle for 12 h, followed by immunofluorescence detection. β-actin and DAPI were used as internal references. All of the experiments were repeated three times.

After translocation into the nucleus, NFAT1 transactivates its downstream target genes, *e.g.*, c-Myc, COX-2, and MDM2, resulting in cancer cell proliferation and migration [31–34]. Therefore, we further examined the effects of JapA on NFAT1 localization in both MCF-7 and MDA-MB-231 cell lines. As shown in Figure 4A, JapA decreased the protein levels of both cytoplasmic and nuclear NFAT1, which could be partially restored by ION and further decreased by CsA. The inhibitory effects of JapA on NFAT1 nuclear localization were also confirmed by an immunofluorescence study in both cell lines. As shown in Figure 4B, JapA treatment decreased the immunostaining of NFAT1 in the nucleus, which led to the repression of its transcriptional activity.

### JapA inhibits the interaction between NFAT1 and the *MDM2* P2 promoter

**Figure 5:**
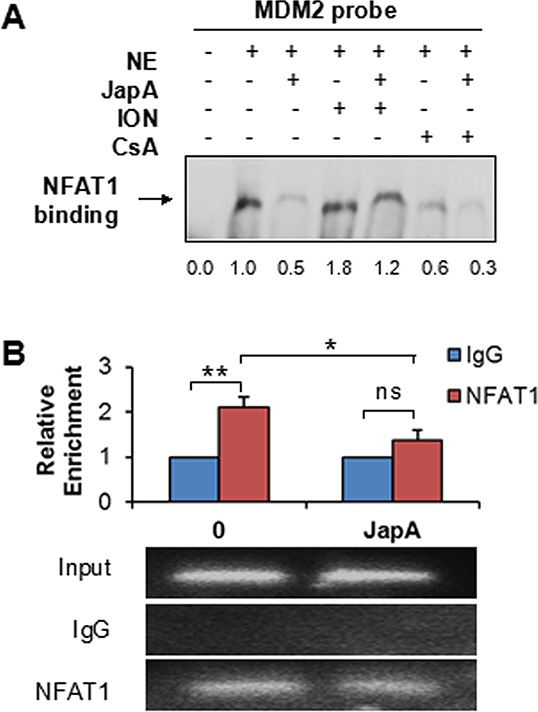
Effects of JapA on the binding of NFAT1 to the *MDM2* P2 promoter **A.** Nuclear extracts from MDA-MB-231 cells treated with JapA (2 μM) in the presence or absence of ionomycin (ION; 4 μM) or cyclosporine A (CsA; 2 μM) were incubated with the *MDM2* probe. An EMSA assay was performed to detect the binding between endogenous NFAT1 protein and the *MDM2* probe. The intensity ratio under each band was obtained by IMAGEJ software analysis normalized on untreated control. **B.** MDA-MB-231 cells were treated with JapA (2 μM) or the vehicle for 24 h. The cell lysates were immunoprecipitated with NFAT1 or IgG antibodies. The DNA bound to the endogenous NFAT1 was eluted and quantified using primers specific for the *MDM2* P2 promoter by PCR. All of the experiments were repeated three times. (*P < 0.05 and **P < 0.01, “ns” denotes “not significant”).

Since NFAT1 activates *MDM2* transcription through direct binding to the *MDM2* P2 promoter [31], we then examined whether JapA affects the interaction between NFAT1 and the *MDM2* P2 promoter. As shown in Figure 5A, an electrophoretic mobility shift assay (EMSA) showed that endogenous NFAT1 from MDA-MB-231 cells was specifically bound to the *MDM2* P2 promoter, and this binding was significantly reduced by JapA treatment. In addition, the nuclear extracts from ION-treated cells showed the enhanced binding of NFAT1 to the *MDM2* probe, whereas simultaneous treatment with CsA inhibited the binding (Figure 5A). We then performed a chromatin immunoprecipitation (ChIP) assay combined with a real-time quantitative PCR analysis. As shown in Figure 5B, endogenous NFAT1 from MDA-MB-231 cells specifically bound the P2 promoter of *MDM2* (*P* < 0.01), and JapA reduced the binding of NFAT1 to the *MDM2* P2 promoter (*P* < 0.05).

### JapA induces the ubiquitination and proteasomal degradation of NFAT1

**Figure 6:**
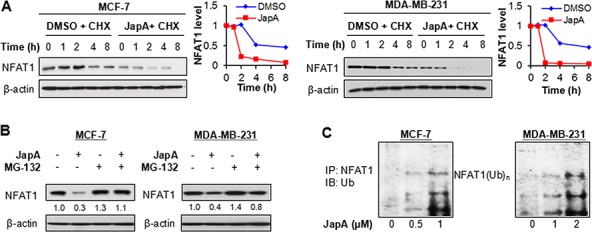
Effects of JapA on NFAT1 protein stability **A.** MCF-7 and MDA-MB-231 cells were treated with JapA or vehicle, followed by exposure to a protein synthesis inhibitor, cycloheximide (CHX, 15 μg/mL). The NFAT1 protein levels were detected by Western blotting at the indicated times after exposure to CHX. Graphs (right) show the quantification of the immunoblotting data. **B.** The cells were treated with JapA or vehicle (1 and 2 μM, respectively, for MCF-7 and MDA-MB-231 cells) for 24 h, then exposed to MG-132 (25 μM), a proteasome inhibitor, for an additional 6 h. The protein levels of NFAT1 were detected by Western blotting. The intensity ratio under each band was obtained by IMAGEJ software analysis normalized on untreated control. **C.** The cells were co-transfected with NFAT1 and ubiquitin plasmids, followed by treatment with JapA for 24 h. Cell lysates were subjected to immunoprecipitation with an NFAT1 antibody. The ubiquitinated NFAT1 was detected using an anti-ubiquitin antibody. All of the experiments were repeated three times.

We next examined the mechanisms responsible for the JapA-induced NFAT1 inhibition. As shown in Figure 6A, JapA increased NFAT1 protein degradation in the presence of a protein synthesis inhibitor, cycloheximide (CHX), shortening the half-life of the NFAT1 protein in both MCF-7 and MDA-MB-231 cell lines. Further studies showed that the NFAT1 reduction induced by JapA was prevented by MG-132 (a proteasome inhibitor) treatment (Figure 6B), which indicated that JapA could induce the proteasomal degradation of NFAT1. These results were supported by the fact that JapA promoted NFAT1 ubiquitination (Figure 6C). Together, JapA promoted the ubiquitination and proteasomal degradation of NFAT1, and thereby inhibited the expression and activity of NFAT1 in breast cancer cells *in vitro* and *in vivo*.

### The cell response to JapA is affected by NFAT1 overexpression and knockdown in breast cancer cells

**Figure 7:**
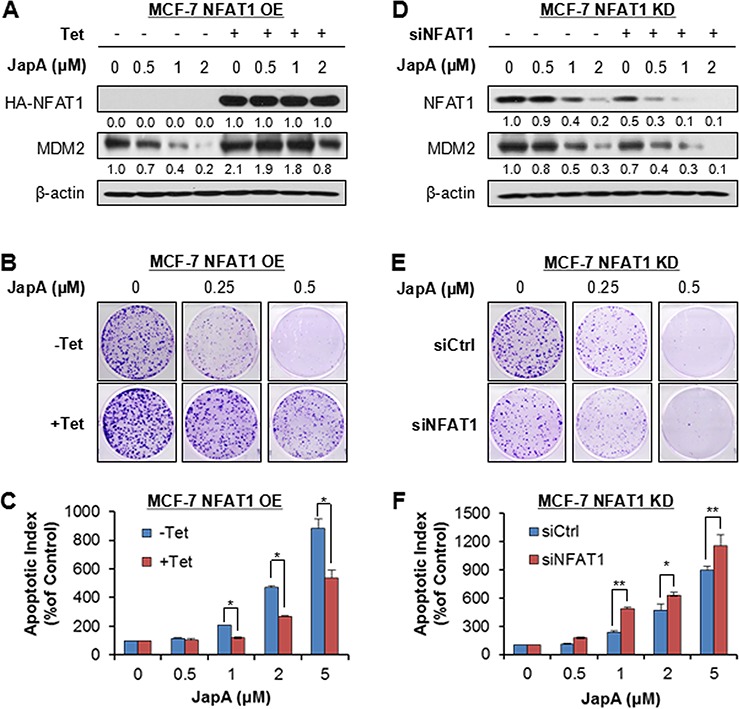
NFAT1 overexpression and knockdown affect the cell response to JapA in breast cancer cells **A, B.** and **C.** The inducible NFAT1 overexpression MCF-7 cells were incubated with (+Tet; 1 μg/mL) or without tetracycline (−Tet) for 24 h and then treated by various concentrations of JapA for (A) 24 h for the expression of NFAT1 and MDM2; (B) 24 h for the colony formation assay; and (C) 48 h for the apoptosis assay. **D, E.** and **F.** MCF-7 cells were transfected with control siRNA or a NFAT1 siRNA for 36 h, followed by treatment with various concentrations of JapA for (D) 24 h for the expression of NFAT1 and MDM2; (E) 24 h for the colony formation assay; and (F) 48 h for the apoptosis assay. The intensity ratio under each band was obtained by IMAGEJ software analysis normalized on untreated control. All assays were performed in triplicate and repeated three times (*P < 0.05 and **P < 0.01).

The importance of NFAT1 and NFAT1-MDM2 pathway in JapA's anti-breast cancer activity was further demonstrated using Tet-on inducible NFAT1 overexpression (OE) and siRNA NFAT1 knockdown (KD) MCF-7 cells. Tet-induced NFAT1 OE upregulated the MDM2 expression (Figure 7A), and reduced the effects of JapA on NFAT1-MDM2 signaling (Figure 7A), colony formation (Figure 7B), and apoptosis (Figure 7C) in MCF-7 cells. In contrast, NFAT1 KD decreased the MDM2 expression levels (Figure 7D), and enhanced the effects of JapA on NFAT1-MDM2 pathway (Figure 7D), colony formation (Figure 7E), and apoptosis (Figure 7F) in MCF-7 cells.

**Figure 8:**
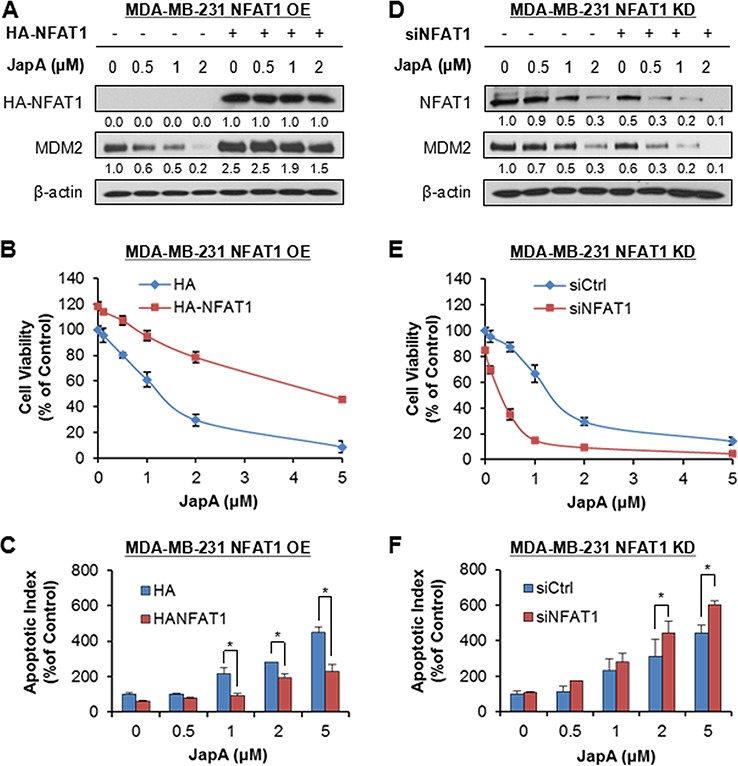
NFAT1 overexpression and knockdown affect the cell response to JapA in breast cancer cells **A, B.** and **C.** MDA-MB-231 cells were transfected with an HA plasmid or an HA-tagged NFAT1 plasmid for 24 h and then treated with various concentrations of JapA for (A) 24 h for the expression levels of HA-NFAT1 and MDM2; (B) 72 h for determination of the cell viability; and (C) 48 h for the apoptosis assay. **D, E.** and **F.** MDA-MB-231 cells were transfected with NFAT1 siRNA or the respective control siRNA for 36 h and then treated with various concentrations of JapA for (D) 24 h for expression levels of NFAT1 and MDM2; (E) 72 h for determination of the cell viability; and (F) 48 h for the apoptosis assay. The intensity ratio under each band was obtained by IMAGEJ software analysis normalized on untreated control. All assays were performed in triplicate and repeated three times (*P < 0.05).

We further demonstrated the importance of NFAT1 and NFAT1-MDM2 pathway in JapA's anticancer activity using NFAT1 OE and KD MDA-MB-231 (p53 mutant) cells. NFAT1 OE in MDA-MB-231 cells activated MDM2 (Figure 8A) and reduced the effects of JapA on NFAT1-MDM2 pathway (Figure 8A), cell viability (Figure 8B), and apoptosis (Figure 8C). However, NFAT1 KD decreased the expression levels of MDM2 (Figure 8D) and enhanced the effects of JapA on NFAT1-MDM2 pathway (Figure 8D), cell viability (Figure 8E), and apoptosis (Figure 8F) in MDA-MB-231 cells.

## DISCUSSION

In the present study, we demonstrated that the newly discovered MDM2 inhibitor JapA inhibits NFAT1 and NFAT1-MDM2 signaling, which plays a critical role in its anticancer activity. Our findings provide evidence for the value of NFAT1 and NFAT1-MDM2 signaling as novel targets in developing effective targeted therapy for human cancers.

We have made several important discoveries in the present study. First, JapA represents a new class of NFAT1 inhibitors that target both NFAT1 and NFAT1-MDM2 signaling. Second, JapA inhibits the expression and activity of NFAT1 in breast cancer cells *in vitro* and *in vivo*. Third, JapA inhibits NFAT1-mediated expression of the transcriptional responsive genes by inhibiting NFAT1 nuclear localization and disrupting NFAT1-DNA binding. Fourth, JapA induces NFAT1 protein ubiquitination and proteasomal degradation. Finally, the inhibition of NFAT1 and NFAT1-MDM2 pathway is critical for JapA's anticancer activity. Our results demonstrated the characterization of JapA as a novel NFAT1 inhibitor and the importance of NFAT1 and NFAT1-MDM2 signaling in JapA's inhibitory effect against human cancer.

Our previous studies have demonstrated that JapA is a selective and potent inhibitor of MDM2 and has novel molecular mechanisms distinct from other MDM2 inhibitors under development [13]. JapA directly binds to MDM2 protein and induces MDM2 autoubiquitination and proteasomal degradation. JapA also inhibits *MDM2* transcription in an NFAT-dependent manner, suggesting that JapA inhibits MDM2 via alternative mechanisms different from the MDM2 inhibitors that target MDM2-p53 interaction and MDM2 itself. It has been reported that the transcription factor NFAT1 activates MDM2, independent of p53 [31]. In the present study, we demonstrated that the inhibitory effects of JapA on *MDM2* transcription depend on the inhibition of NFAT1, regardless of the p53 status of the cells.

NFAT proteins (NFAT1-NFAT5) are a group of inducible transcription factors and have been implicated in many biological processes, including cancer development and progression [24–26]. We and other investigators have suggested NFAT as a potential molecular target for cancer therapy [24–26]. NFAT1, also named NFATp or NFATc2, is overexpressed and hyperactivated in human cancers, including breast cancer [27–30]. Aberrant activation of NFAT1 signaling results in the upregulation of genes associated with tumor growth and metastasis, such as MDM2 and c-Myc [24–26]. Recent evidences have suggested that NFAT1 plays a critical role in breast cancer development and progression. It has been found that NFAT1 promotes breast cancer cell motility and invasion by regulating COX-2 and glypican-6 [30, 34–35]. NFAT1 also functions as a critical factor in Akt/protein kinase B (PKB) and Glycogen synthase kinase 3β (GSK3β) signaling pathways to regulate the breast cancer metastasis [36]. In addition, NFAT1 promotes intratumoral neutrophil infiltration by regulating interleukin-8 (IL8) expression in breast cancer [37]. NFAT1 and signal transducer and activator of transcription 5 (Stat5) signaling cascades have been found to antagonize each other in breast cancer, which may affect breast cancer initiation, growth, and metastasis [28]. Importantly, the constitutive activation of NFAT1 signaling has been observed in diagnostic breast cancer cases, and it is essential to the tumorigenic and metastatic potential of breast cancer cells [38]. Therefore, targeting NFAT1 is a promising strategy for developing novel and effective anti-breast cancer therapy.

NFAT1 exerts its oncogenic functions in human cancer through calcineurin-dependent and –independent pathways [24–26]. Once activated, calcineurin directly dephosphorylates NFAT1 protein, leading to its nuclear translocation and the transactivation of its target genes [24, 26]. The balance between nuclear import and export of the NFAT1 protein is also regulated by various kinases, such as GSK3β, casein kinase 1 (CK1), and protein kinase A (PKA), independent of calcineurin [24]. Several strategies have been demonstrated to develop novel NFAT inhibitors. First, targeting the upstream regulators of NFAT proteins, such as calcineurin and GSK3β, to inhibit NFAT protein dephosphorylation and nuclear translocation; second, directly targeting NFAT proteins themselves to inhibit NFAT expression and nuclear translocation; and third, blocking NFAT-DNA binding to inhibit NFAT transcriptional activity. Thus far, most of the NFAT inhibitors under development have been designed by following the first strategy, such as VIVIT peptides [39–40], PxIxIT peptides [41], CsA [42–44], FK-506 [44–45], and NCI3 [46].

Increasing evidences have suggested that different NFAT isoforms even have opposite roles in the same cancer type. For example, the overexpression and hyperactivation of NFAT1 and NFAT2 are frequently observed in human cancer and contribute to cancer development, progression, and metastasis [24, 26, 31, 47–49]. However, NFAT3 has been reported to be specifically expressed in estrogen receptor α positive breast cancer cells and reduce cancer cell motility by inhibiting the expression of Lipocalin 2 [50]. Up to date, most of the NFAT inhibitors have been developed to nonspecifically inhibit all calcineurin-responsive NFAT isoforms no matter whether they exert their roles as an oncogene or a tumor suppressor, resulting in suboptimal efficacy against human cancer and unexpected side effects on human immune system. Several NFAT inhibitors that target either oncogenic NFAT isoforms, such as helenalin [51] and zoledronic acid [52], or NFAT-DNA binding, such as imperatorin [53] and digitoxin [54] have shown significant anticancer activities *in vitro* and *in vivo*. The present study reports, for the first time, a novel NFAT1 inhibitor JapA that targets both oncogenic NFAT1 and NFAT1 interaction with the *MDM2* P2 promoter.

We have demonstrated that JapA is a novel NFAT1 inhibitor. Several assays have been performed to demonstrate the specificity of JapA in targeting NFAT1 and NFAT1-MDM2 pathway in breast cancer cells. First, the inhibitory effects of JapA on the expression of NFAT1 and its target genes, MDM2, c-Myc and COX-2, were shown in human breast cancer cells *in vitro* and breast xenograft tumors *in vivo*. Second, the inhibitory effects of JapA on NFAT1-mediated MDM2 activation were demonstrated using breast cancer cells with NFAT1 OE or KD, as well as other pharmacological inhibitors and activators of calcineurin-NFAT signaling. Third, the inhibitory effects of JapA on NFAT1 nuclear localization and NFAT1-DNA binding were demonstrated using immunostaining, EMSA and CHIP assays. NFAT1 activates MDM2 through interacting with the *MDM2* P2 promoter but not the P1 promoter, while our results have shown that JapA inhibits the binding of NFAT1 to *MDM2* P2 promoter, independent of p53. Importantly, the positive regulation of MDM2 by NFAT1 has been observed in human hepatocellular carcinoma tissue samples, regardless of the p53 status of the tumors [13]. Since JapA represents a new class of MDM2 inhibitors that inhibit both MDM2 and its activator NFAT1, it could be more effective than other NFAT1 or MDM2 inhibitors in treating tumors with high expression levels of both NFAT1 and MDM2. Moreover, its excellent efficacy for treating tumors with both wild-type p53 and non-functional p53 is also expected. Fourth, the JapA-induced NFAT1 protein destabilization was attributed to NFAT1 ubiquitination and proteasomal degradation, which was elucidated using protein synthesis inhibitor and proteasome inhibitor and determined by an ubiquitination assay. Although it has been reported that the NFAT1 ubiquitination is mediated by MDM2 through its E3 ubiquitin ligase activity [30], more direct evidence is still needed for the role of MDM2 in JapA-induced NFAT1 protein degradation.

We have further demonstrated that NFAT1 and NFAT1-MDM2 pathway play a critical role in JapA's inhibitory effects against human cancer, as indicated in NFAT1 OE and KD MCF-7 (p53 wild-type) and MDA-MB-231 (p53 mutant) breast cancer cell lines. Despite these results, there is no direct evidence that JapA just specifically inhibits NFAT1 and further studies on its effects on other NFAT isoforms are needed. The determination and validation of the direct binding of JapA to NFAT1 would help address these issues partially. Moreover, it is possible that JapA may have other molecular targets that are important for its anticancer activity; further exploration of additional targets also needs to be considered, such as MDMX and other oncogenic NFAT isoforms.

Of note, we have shown the *in vivo* efficacy of JapA and its safety profile at two dose levels (15 and 30 mg/kg/day) in breast cancer xenograft models [13]. We have found that JapA effectively inhibits the tumor growth and the protein expression of MDM2 and NFAT1 *in vivo*, without inducing any apparent host toxicity. It is expected that JapA at these dose levels is safe and effective in treating other tumors with high expression levels of MDM2 and NFAT1, regardless of p53 status of the tumors. However, the toxicological profile of JapA needs to be further characterized using more clinically relevant animal models, especially syngeneic models, since NFAT1 plays a critical role in human immune system and developmental programs. The effects of JapA on the expression and activity of NFAT1 should be further examined in both immune cells and tumor cells to determine if JapA affects the immune system or it is selective for tumor cells.

In conclusion, our study demonstrates that JapA inhibits the expression and activity of oncogenic NFAT1 through inhibiting NFAT1 nuclear localization and NFAT1-DNA binding and promoting NFAT1 ubiquitination and proteasomal degradation, which contributes to the anticancer activity of JapA. These results indicate that JapA is a novel anticancer drug candidate with a dual-targeting mechanism and provide new insights into the future development of novel dual inhibitors of NFAT1 and MDM2 for cancer therapy. However, it is tempting to speculate that the unique inhibitory effects of JapA on NFAT1-MDM2 pathway and its efficacy and toxicological profile should be further explored for the elucidation of uncharacterized mechanisms of action, as well as the development of safe and effective anticancer therapy.

## MATERIALS AND METHODS

### Cell culture

Human breast cancer (MCF-7, MDA-MB-231, and MDA-MB-468) and non-malignant epithelial (MCF-10A) cells were obtained from the American Type Culture Collection (Rockville, MD). Human mammary luminal epithelial (HMLE) cells were obtained from Zen-Bio, Inc. (Research Triangle Park, NC). The MCF-7 p53^−/−^ and the inducible NFAT1 OE MCF-7 cell lines were established previously [31, 55–56]. HMLE cells were grown in mammary luminal epithelial cell growth medium (Zen-Bio, Inc.). Other cell lines were cultured as reported by us earlier [13].

### Chemicals, reagents, antibodies, plasmids, and siRNA

JapA was obtained as previously reported [13], with the purity being > 95% as confirmed by IR, ESI-MS, NMR, and HPLC/MS^n^ [16–17]. All chemicals and solvents were of the highest analytical grade available. The anti-human NFAT1 (1/NFAT-1) antibody was from BD Biosciences (San Jose, CA). The anti-human COX-2 (C-20) and c-Myc (0.N.222) antibodies were from Santa Cruz Biotechnology Inc. (Dallas, TX). The anti-human MDM2 (Ab-2) antibody was from Calbiochem (Billerica, MA). The anti-human ubiquitin (6C1) and β-actin (AC-15) antibodies were from Sigma (St. Louis, MO). Goat anti-mouse IgG (H+L) and goat anti-rabbit IgG (H+L) were obtained from Bio-Rad (Hercules, CA). The human full-length *MDM2* P2 promoter reporters were kind gifts from Dr. J.P. Blaydes (Southampton General Hospital, UK). Vectors expressing HA-NFAT1, CA-NFAT1, and DN-NFAT were kindly provided by Dr. C.W. Chow (Yeshiva University). NFAT1 siRNA or control siRNA were from Thermo Scientific (Rockford, IL). Both plasmids and siRNAs were transfected into cells using the same protocols as we reported previously [57].

### Assays for cell viability, colony formation and apoptosis

The cells were treated with various concentrations of JapA, and the cell viability, cell colony formation and apoptosis assays were performed as described previously [58–59].

### Real-time quantitative PCR, immunoblotting, and luciferase assay

For real-time quantitative PCR analysis, total RNA was extracted using the Trizol reagent (Invitrogen, Grand Island, NY) and analyzed as described previously [59–60]. For immunoblotting assay, cell lysates and tissue homogenates were prepared in NP40 lysis buffer containing protease inhibitors (Sigma, St Louis, MO). The protein concentration was estimated using the Bradford reagent (Bio-Rad, Hercules, CA). The expression levels of various proteins were examined as described previously [58–59]. For luciferase assay, cells were co-transfected with full-length human *MDM2* P2 promoter vector with the Renilla luciferase reporter as an internal control [13]. After exposure to JapA for 24 h, the luciferase activity of the *MDM2* promoter reporters was determined using the Dual-Luciferase Reporter Assay System (Promega, Madison, WI).

### Ubiquitination assay

Cells were co-transfected with NFAT1 and ubiquitin plasmids and treated with various concentrations of JapA. Cell lysates were immunoprecipitated with anti-NFAT1 antibody, and the bound proteins were purified with protein G-Sepharose beads (Sigma, St Louis, MO), resolved on SDS-PAGE, and detected by an anti-ubiquitin antibody [13].

### Electrophoretic mobility shift assay (EMSA)

MDA-MB-231 cells were treated with JapA (2 μM), ION (4 μM), and/or CsA (2 μM) for 24 h, and nuclear factions were extracted using the NE-PER Nuclear and Cytoplasmic Extraction Kit (Thermo Scientific, Rockford, IL). The NFAT1 DNA-binding activity was measured as described previously [31]. The prepared nuclear extracts were preincubated with 1 μg of poly-(dI:dC) (Thermo Scientific, Rockford, IL) and then reacted with a biotin-labeled MDM2 probe at room temperature for 30 min. The sequences of the biotin-labeled probe were MDM2 forward, 5′-gcaggttgactcagcttttcctcttgagctggtcaagttca-3′ and MDM2 reverse, 5′-tgaacttgaccagctcaagaggaaaagctgagtcaacctgc-3′.

### Chromatin immunoprecipitation (ChIP) assay

MDA-MB-231 cells were treated with DMSO or JapA (2 μM) for 24 h, then the chromatin immunoprecipitation (ChIP) assay was performed in the treated cells as described previously [31]. The specific primer pairs used were: 5′-cccccgtgacctttaccctg-3′ and 5′-agcctttgtgcggttcgtg-3′ for qualitative or quantitative PCR amplification of the responsive element of the *MDM2* promoter.

### Cell immunofluorescence assay and immunohistochemistry

For immunofluorescence assay, cells were seeded on coverslips in a 12-well plate at a density of 10,000 cells/well, allowed to attach overnight, and treated with JapA for 12 h. The cells were then processed for immunofluorescence detection as reported [58]. For the immunohistochemical studies, the fresh tumor tissues were dissected from the mice bearing both MCF-7 and MDA-MB-231 xenograft tumors [13]. These breast cancer xenograft models were developed by following the animal study protocols that have been approved by the Institutional Animal Use and Care Committee of the Texas Tech University Health Sciences Center. The dose, dose frequency, and administration route were chosen based on the previous studies [13, 18, 20]. The dissected tumor tissues were fixed and stained for the protein expression and location of NFAT1 following the reported protocols [13].

### Statistical analysis

The data are expressed as the means ± SEM from at least three independent experiments. Two-sided *t*-test was used for comparisons between two groups. *P* < 0.05 was considered as statistically significant.

## Abbreviations

CA-NFAT1: constitutively-activated NFAT1
ChIP: chromatin immunoprecipitation
CHX: cycloheximide
CK1: casein kinase 1
COX-2: cyclooxygenase 2
CsA: cyclosporine A
DN-NFAT: dominant-negative NFAT
EMSA: electrophoretic mobility shift assay
GSK3β: glycogen synthase kinase 3β
HMLE: human mammary luminal epithelial cells
IL8: interleukin-8
ION: ionomycin
KD: knockdown
MDM2: mouse double minute 2
NFAT: nuclear factor of activated T cells
OE: overexpression
PKA: protein kinase A
PKB: protein kinase B
Stat5: signal transducer and activator of transcription 5
